# Supplementary material for: Barriers to Radiotherapy Access in Sub-Saharan Africa for Patients with Cancer: A Systematic Review
Source: Int J Environ Res Public Health. 2024 Nov 30;21(12):1597. doi: 10.3390/ijerph21121597 (PMC11675486; doi:10.3390/ijerph21121597)
Supplement: Supplementary file 1 [file ijerph-21-01597-s001.zip › ijerph-3295920-supplementary.pdf]

BARRIERS REPORTED								CONCLUSION
1.		Socioeconomic Status					Life Expectancy	Multilevel interventions are needed to ensure access, availability, and affordability of a minimum standard of care for breast cancer patients in sub-Saharan Africa.
2. Treatment Centre Characteristics						Lack of Understanding/ Awareness		Reducing wait time across this care continuum is needed to improve easy access to healthcare, quality care, survival and patient outcomes, as many patients still face longer wait times for diagnosis and treatment of lung cancer than recommended in several healthcare settings. A multidisciplinary team approach will help to reduce wait time and ensure that all patients receive appropriate care. Interventions are needed to address delays in lung cancer care in LMICs. Health-care providers at all levels of care should be educated and equipped with skills to identify lung cancer symptoms and perform or refer for appropriate diagnostic tests
3. Treatment Centre Characteristics								A diagnosis of cervical cancer introduces significant socioeconomic disruptions in a woman and her family life. Cervical cancer causes disability, impairs the woman and her family productivity and exacerbates levels of poverty in the home. High and expensive out-of-pocket expenditure on investigations, treatments and transport costs further compound the socioeconomic burden.
4. Treatment Centre Characteristics								The findings of our study show the critical role of family support in the management of breast cancer; therefore, family members should be encouraged to give breast cancer patient the necessary support to help them manage their sick

											role behavior since their illness has no cure.
5.	Treatment Centre Characteristics	Waiting Times		Socioeconomic Status							Several assignments are waiting for the policy makers, the professionals, the communities and other concerned bodies to combat the alarmingly growing burden of cancer in Ethiopia. Escalating the awareness of the general population about cancer, expanding well-developed diagnostic and treatment centers, and producing well-trained competent oncology professionals are the forefront challenges in combating cancer in Ethiopia.
6.	Treatment Centre Characteristics			Socioeconomic Status		Cultural Beliefs			Lack of Understanding/ Awareness		Operational level healthcare professionals in northern Uganda reported several practical challenges facing cervical cancer care that influence their decisions, management goals and practices. The challenges and proposed remedies can inform targeted interventions for early detection, management, and control of cervical cancer in Uganda.
7.			Distance to Radiotherapy Centre	Socioeconomic Status	Age	Cultural Beliefs					Generally, knowledge inadequacy of BC and its early detection measures continue to be one of the most important factors in determining women's attitude towards BC screening and treatment. Understanding the benefits of early detection and presentation of BC among women was poor across all studies. Sources of information, knowledge of early detection measures, sociocultural beliefs and traditions revealed women's perceptions of BC. Societal traditions and beliefs play an important role in women's BC perceptions, screening measures and treatment. The findings of this study prompt educational campaigns among African women to enhance their

							knowledge of BC, benefits of early detection and treatment.
8.	Treatment Centre Characteristics						The public sector faces infrastructural and key personnel shortages. Therefore, infrastructural and human resource challenges should be prioritised by policy-makers and administrators. Additionally, the vital contributions of psycho-social professionals should be incorporated in policies and programs supporting cancer care, in order to improve the cancer patient care. The results of this study may help decision makers to further improve cancer care in South Africa.
9.	Treatment Centre Characteristics						These findings highlight the need for interventions at the patient, provider, and health system levels to improve cancer care quality and outcomes in Botswana. Results also suggest that widespread cancer education has potential to promote early diagnosis through family and community networks. Identified barriers and facilitators suggest that interventions to improve community education and access to diagnostic technologies could help improve cancer outcomes in this setting.
10.	Treatment Centre Characteristics						This study opens an in-depth examination of the stigma associated with breast cancer and the impact it has on cancer care engagement in Uganda. The findings from this study are consistent with research on barriers to early detection in other Sub-Saharan African countries. We hope that this study serves to encourage further research with aims to improve early detection, treatment engagement, and treatment completion for women with breast cancer in low-resource settings. The growing burden of mortality

										associated with breast cancer worldwide can be stemmed with increased resources for cancer care and evidence-based interventions, which include accounting for key psychosocial barriers, such as stigma.
11.	Treatment Centre Characteristics	Waiting Times	5 to Radiotherapy Centre							The findings revealed that Limpopo province has a shortage of high-technology medical equipment, poor coordination, and a lack of oncological and allied expertise. Governments should ensure that patients receive the care required as stated in the constitution to navigate cancer care pathways to improve patient health outcomes, particularly in rural areas where care is fragmented and poorly financed. Recommendations to support oncology patients involve psychosocial work and palliative care of the multidisciplinary teams to be put forward. The identified barriers regarding oncology care may contribute to changing the departmentsâ€™ outlook and effective functioning by including interdisciplinary oncology teams at all levels of care.
12.	Treatment Centre Characteristics	Waiting Times	Distance to Radiotherapy Centre	Socioeconomic Status	Cultural Beliefs	Patient preferences				Hundreds of thousands of patients with cancer in LMICs continue to live in countries without access to RT in their own country. This extreme form of global health inequity requires urgent and decisive action, the success of which depends on the integration of international and local efforts.
13.			Distance to Radiotherapy Centre							Cancer patients in SSA travel considerable distances to access radiotherapy services. HFRT decreases patient-related costs and time expenditures, which may increase radiotherapy access and alleviate the growing burden of cancer in the region
14.		Waiting		Socioecono	Cultural	Patient				Pediatric oncology services in Nigeria are

Times	mic Status	Beliefs	preferences				still grappling with weak human capital, poorly developed infrastructure, weak regional and national referral systems, and poor support services.
15. Treatment Centre Characteristics							The findings support that economic hardships, fear, and scarcity of cancer treatments/equipment are critical in limiting access to breast cancer care. Sustainable strategies aimed at scaling-up breast cancer care in the region are necessary. The results also highlight the need for reduced treatment cost and aggressive educational campaigns across healthcare facilities and the local communities.
16. Treatment Centre Characteristics				Lack of Referr al			Considerable barriers to the early identification and treatment of lung cancer exist. Finding solutions to overcome both individual and health-system level obstacles to lung cancer screening and management are vital to facilitate early identification and treatment, and to improve survival. Furthermore, research on inexpensive biomarkers for asymptomatic disease detection, the introduction of diagnostic imaging tools that utilise artificial intelligence to compensate for inadequate human resources and improving clinical integration across all levels of the healthcare system are essential.
17. Treatment Centre Characteristics							The present review shows that treatment delay among women with breast cancer in sub-Saharan Africa is influenced by many related health system factors. Policy makers in sub-Saharan Africa need to tackle the financial accessibility to breast cancer treatment by adequate universal health coverage policies and reinforce the clinical competencies for health workers

						to ensure timely diagnosis and appropriate care for women with breast cancer in this region.
18.	Treatment Centre Characteristics					Our review indicates that diagnostic and treatment intervals among women with breast cancer in sub-Saharan Africa are influenced by many health system related factors. When women manage to overcome their fear, lack of knowledge, socioeconomic and cultural conditions, of knowledge, socioeconomic and cultural conditions, they also end up being challenged by overwhelming health system factors that they cannot cope with. Our review sheds light on the underlying factors that explain the longer time intervals and health system challenges women face in terms of financial and geographical access to care, diagnostic errors, inappropriate management and lack of an adequate cancer health policies.
19.	Treatment Centre Characteristics					We identified stigma and other multilevel modifiable patient and health system factors associated with treatment fidelity to guideline-concordant breast cancer treatment. The facilitators provide opportunities for leveraging existing strengths within the Botswana context to design implementation strategies to increase treatment fidelity to guideline-concordant breast cancer therapy. However, PWH experienced unique barriers, suggesting that interventions to address treatment fidelity may need to be tailored to individual circumstances such as comorbidities.
20.	Treatment Centre Characteristics					This mixed method study using both quantitative data and qualitative data coming from head and neck cancer

											patients in a low-income country is a first step towards the understanding of the access to care for this specific patient population. Future work is needed to further define cost-effective strategies for early detection of head and neck cancer in low- and lower-middle income countries.
21.	Treatment Centre Characteristics		Distance to Radiotherapy Centre	Socioeconomic Status		Cultural Beliefs	Patient preferences				In conclusion, clinicians in SSA have basic tools to improve breast cancer outcomes. Progress in domains such as radiotherapy and systemic therapy is proceeding rapidly, whereas in other domains such as imaging, it remains slow. Late-stage presentation and high cost burden are persistent obstacles to effective breast cancer management. Opportunities to further expand skilled workforce, including pathology, medical, and surgical oncology specialties, should translate into improved quality of care.
22.				Socioeconomic Status	Age						This is the first qualitative systematic review undertaking a narrative synthesis of factors contributing to the delays in diagnosis and treatment of different cancer types in SSA. Although 39 studies were identified over the 25-year period, these predominantly focused on two main tumour types, breast and cervix, with no studies identified for skin cancers such as Kaposi sarcoma and only a single study for prostate and lung across the whole of SSA, which is insufficient. The studies identified do provide different perspectives about the complex interlinked factors that contribute to the barriers to timely diagnosis and treatment of cancer in SSA. The results have highlighted the need for health policy makers and researchers to understand people's help-seeking behaviour and to address the economic

									barriers to receiving care, as well as the social-cultural factors that perpetuate stigma resulting from a lack of knowledge and awareness of cancer. In addition, health personnel need adequate diagnostic and treatment resources as well as training in the management of cancer, with clear pathways of referral between primary/community care and specialist facilities.
23.	Treatment Centre Characteristics	Waiting Times			Patient preferences				The barriers to cancer care were multifactorial and related to local cultural, financial, and geographic characteristics. Understanding these barriers may enable us to reintegrate defaulting patients into the health care system. The findings of this study can be used in piloting cancer navigation programs across sub-Saharan Africa and the rest of the world.
24.			Distance to Radiotherapy Centre						We identified areas needing comprehensive cervical cancer care infrastructure, human resources, and training programs. There are major gaps in access to radiation oncologists and trained gynecologic oncologists in Africa.
25.			Distance to Radiotherapy Centre						The challenges in chemotherapy included unavailability and affordability, low survival rates, treatment interruption due to stock-outs as well as late presentation. Major challenges on radiotherapy were unavailability of radiotherapy, treatment interruption due to financial constraints, and machine breakdown and low quality of life. A gap in understanding the status of CC management in SSA has been revealed by the study implying that, without full knowledge of the extent of CC management, the challenges and opportunities, it will be difficult to reduce infection, improve treatment and palliative

										care. Research projects assessing knowledge, attitude and practice of those in immediate care of girls at vaccination age, situational analysis with health professionals and views of patients themselves is important to guide CC management practice.
26.	Treatment Centre Characteristics			Socioeconomic Status	Cultural Beliefs	Patient preferences	Lack of Referral			Barriers to care at a radiotherapy center in a low- and middle-income country (LMIC) have previously not been well characterized. These findings can be used to inform efforts to expand the availability of radiotherapy and improve current treatment capacity in Nigeria and in other LMICs
27.	Treatment Centre Characteristics		Distance to Radiotherapy Centre							Robust research to inform policy on the barriers to quality cancer care in SSA is absent. The focus of most research is on breast and cervical cancers. Research outputs are from few countries. It is imperative that we investigate the complex interaction of these factors to build resilient and effective cancer control programmes.
28.			Distance to Radiotherapy Centre							Down-staging strategies are only useful if the continuum of breast cancer care is warranted for the majority of patients.
29.	Treatment Centre Characteristics			Socioeconomic Status	Cultural Beliefs	Patient preferences				With rapidly rising cancer incidence in LMICs, efforts to improve early cancer diagnosis and treatment through system-level interventions and individual behavioural interventions are critical to reduce cancer mortality. Interventions must address major barriers to medical help-seeking for symptoms and decisions to access healthcare for diagnosis and treatment in LMICs by raising cancer awareness, modifying negative beliefs and addressing cultural barriers such as TCAM

									use and barriers for women.
30.			Socioeconomic Status	Cultural Beliefs	Patient preferences				Nigeria faces a gross shortage of radiation oncologists and other personnel, with under supply of megavoltage machines. Careful planning is required to allocate adequate resources for manpower development and purchase of equipment. There is need of additional radiotherapy centers in all geopolitical zones and further strengthening of existing ones.
31.		Distance to Radiotherapy Centre	Socioeconomic Status		Patient preferences				Creating breast cancer awareness alongside clear guidelines on accessing screening and treatment infrastructure is critical. It was evident, a diagnosis of breast cancer or lump brings unexpected confrontation with mortality; fear, pain, cultural barriers, emotional and financial distress. Without clear referral channels to enable those with suspicious lumps or early stage disease to get prompt diagnosis and treatment, then well-meaning awareness will not necessarily contribute to reducing morbidity and mortality.
32. Treatment Centre Characteristics									Based on the investigation undertaken to identifying the main issues encountered by the cervical cancer patients in accessing radiation therapy, our data confirmed that the problems which are detrimental to achieving a good quality of life for the participants despite the availability of good treatment regimens and improved ways to treat the disease are due to their socio-economic background which deprive them easy access to their treatment facilities due to the geographical distance and its cost implications.
33.	Waiting Times	Distance to				Lack of	Lack of Understanding/A		In LMICs, children with cancer face multifactorial barriers to access timely

			Radiotherapy Centre				Referral	wareness		cared for across the entire continuum of care. We reviewed the current evidence of the key driver of delays in care and found geographic, social, financial, health system, and health policy barriers that limit access to cancer care in LMICs. Based on these findings, we proposed a Three-Delay frame work that can be used as a policy decision tool to guide financing streams and interventions to improve timely care and survival rates for children with cancer in LMICs. We encourage governments and other public health leaders to use this tool as a template to explore their unique barriers and limitations.
34.				Socioeconomic Status						Sub-Saharan African represents a diverse region with barriers to RT on the basis of funding, available technology and staff and community populations
35.	Treatment Centre Characteristics	Waiting Times	Distance to Radiotherapy Centre	Socioeconomic Status	Cultural Beliefs	Patient preferences				Sub-Saharan Africa represents a diverse region with barriers to RT that differ on the basis of funding, available technology and staff, and community populations. Although long-term solutions must focus on building capacity by increasing the number of treatment machines and providers, short-term improvements should be implemented, such as interim housing for traveling patients, increased community education to reduce late-stage diagnoses, and use of virtual visits to avoid travel.
36.	Treatment Centre Characteristics	Waiting Times		Socioeconomic Status	Cultural Beliefs	Patient preferences				Geographic access to cervical cancer screening, and precancer treatment is relatively widespread across The Gambia, but targeted expansion in line with the countryâ€™s â€œStrategic Planâ€ would improve access for central and eastern Gambia. The availability of treatment

										services for invasive cancer is limited, and establishing radiotherapy in the country should continue to be prioritized.
37.	Treatment Centre Characteristics	Waiting Times	Distance to Radiotherapy Centre							This study revealed few variations in the participation of women in cervical cancer screening and treatment explained only by religious affiliations and usage of health facilities. Strengthening of health education in communities including churches and universal healthcare coverage are recommended strategies to improve uptake of screening and treatment of cervical cancer
38.	Treatment Centre Characteristics	Waiting Times	Distance to Radiotherapy Centre	Socioeconomic Status	Cultural Beliefs					Women with cervical cancer had a higher probability of being diagnosed with advanced stage if they had a lower degree of education, old age, being single and HIV infection. These women should be targeted for regular cervical cancer screening. As one method of reducing the burden of advanced diagnosis of cervical cancer, health education programs and awareness campaigns to raise awareness among women about gynaecological symptoms should be provided. Cervical cancer screening and management of cervical cancer should be integrated with routine HIV care and treatment programs. Further research on the health system barriers for delayed cervical cancer is recommended.
39.	Treatment Centre Characteristics									Despite availability of dedicated pediatric oncology treatment their ability to come to and remain at a central tertiary care facility for treatment is limited. We suggest that the extensive system now in place in most of sub-Saharan Africa that sustains life-long antiretroviral therapy for children with human immunodeficiency virus (HIV) infection be adapted for pediatric cancer

											treatment to improve outcome.
40.	Treatment Centre Characteristics										Waiting time to radiotherapy initiation for women with cervical cancer is considerably long in Addis Ababa and it is associated with disease progression and poor survival. Furthermore, nearly 1 in 10 cervical cancer patients deceased while waiting for receipt of radiotherapy. These findings reinforce the need for coordinated efforts between the local government and national and international private and public health agencies to expand the availability of radiotherapy services in the country in order to mitigate the undue high burden of morbidity and mortality associated with the disease.
41.	Treatment Centre Characteristics			Socioeconomic Status	Age	Cultural Beliefs					In conclusion, our study reported large variations in participation among different African countries. There were substantial differences between African countries in the availability of screening programs as a result of the resources allocated to healthcare development. Radiation capacity is the most limited treatment modality available, followed by the lack of gynecologists or gynecologic oncologists who can provide surgical care for women with cervical cancer. This information is critical for public health educators and policymakers aiming to improve the outcomes among women with cervical cancer in Africa.
42.			Distance to Radiotherapy Centre	Socioeconomic Status	Age	Cultural Beliefs			Lack of Understanding/Awareness		Although delays caused by patient factors are reported extensively, patients overcame these barriers in the hopes of curative treatment. However, staff and equipment malfunctions prevented patients from receiving timely radiotherapy. Policies aimed at addressing machine maintenance, health care worker

											satisfaction, and the aging power grid in Nigeria must be implemented in the future to strengthen the health care system to care for patients with cancer.
43.				Socioeconomic Status	Age						Nearly all patients surveyed experienced a delay in obtaining cancer care. In a setting where care is provided without charge, cancer type and male sex were more important predictors of delays than socioeconomic factors.
44.				Socioeconomic Status		Cultural Beliefs					The study findings compare well with those of a study done in Nigeria by Abiodun et al which found only 2.3% of respondents knew of Human Papillomavirus as a cause of cervical cancer. Reducing cost of treatment, increasing knowledge on cervical cancer as well as introducing comprehensive treatment facilities regionally may increase access and therefore utilization of health care services and thereby improve outcomes for this disease
45.	Treatment Centre Characteristics			Socioeconomic Status							The MDT-coordinated care model allows for systematic benchmarking of the patient treatment cascade. Barriers to timely treatment exist for this cohort in treatment. Interventions to accelerate the timing of the radiation oncology care cascade may improve clinical outcomes in this LMIC setting
46.				Socioeconomic Status		Cultural Beliefs			Lack of Understanding/Awareness		This study showed that the most significant factors affecting adherence were having a tertiary level of education, being married; being within 40-49 years of age; early stage cervical cancer; being treated for not more than 28 days and having less side effects showed better adherence treatment. The recommendations of this study are: close monitoring of unmarried and divorced

										patients; with low levels of education and patients below the age of 39 years and above 60 years. Dose and treatment protocol optimization should be adopted and adapted. Further research studies should done to ascertain the reasons why certain age groups show high adherence to radiotherapy than others.
47.	Treatment Centre Characteristics	Waiting Times	Distance to Radiotherapy Centre	Socioeconomic Status	Cultural Beliefs	Patient preferences	Lack of Referral	Lack of Understanding/Awareness		This study shows that radiotherapy waiting time in LMICs like Nigeria is causing a lot of delay in receiving radiotherapy and is longer than international best-practice targets. Urgent steps need to be taken to address the problems of infrastructure, insufficient radiotherapy professionals, poor care coordination and out-of-pocket payments to minimise the delays so that they are as short as reasonably achievable.
48.			Distance to Radiotherapy Centre	Socioeconomic Status						The most significant fears around breast cancer were related to treatment modalities and adverse effects rather than transport, financial, or work concerns. Young age and job insecurity were predictive of increased fears. Education about treatments has a key role to play in improving access to breast cancer care in South Africa.
49.	Treatment Centre Characteristics									Financial barriers limit the ability of women, especially the poorest SES group, to utilize screening and treatment services for early diagnosis and treatment of breast cancer. Interventions that will improve financial risk protection for women with breast cancer or at risk of breast cancer are needed to ensure equitable access to screening and treatment services
50.	Treatment Centre Characteristics		Distance to Radiother	Socioeconomic Status	Cultural Beliefs					Our findings suggest that factors that are amenable to intervention concerning breast cancer awareness and health care

			apy Centre							access, rather than intrinsic tumour characteristics, are the strongest determinants of stage at diagnosis in Nigerian women.
51.	Treatment Centre Characteristics									Patients presenting with cervical cancer to QECH were young, with a high prevalence of HIV, and late stage disease. The lack of pathological and surgical capacity and the absence of radiotherapy severely limited the possibility of curative treatment. Access to quality palliative care remains an important component of management in low resource settings. Improving awareness of cervical cancer in the community, and better recognition and management within the health service, are important in reducing the cancer burden for women in Malawi.
52.	Treatment Centre Characteristics		Distance to Radiotherapy Centre	Socioeconomic Status	Cultural Beliefs	Patient preferences				Radiotherapy is likely underutilized for women with breast cancer, even in a setting with public sector availability. Exploring patient-level factors that influence adherence to care may provide clinicians with better tools to support adherence and improve survival. Greater investment is needed in multidisciplinary, multi-modality care for breast cancer in SSA.
53.	Treatment Centre Characteristics		Distance to Radiotherapy Centre		Cultural Beliefs		Lack of Referral	Lack of Understanding/Awareness		Challenges faced by cancer patients in Uganda result in enormous delays in initiation and continuÂation of cancer treatment. These challenges are often a result of the poor social-economic status of the patients; inadequate infrastructure for cancer care; and inefficiencies in the health care system.
54.	Treatment Centre Characteristics		Distance to Radiotherapy Centre							Long patient delay and advanced stage diagnosis of breast cancer are higher in our study. Travel distance of 5 km, rural residence, no history of any breast

										problem before, having no lump/swelling in the arm pit, a painless lump in the breast, and being illiterate were important predictors for patient delay. Therefore, public awareness programs about breast cancer should be designed to prevent patient delay in presentation and to promote early detection of cases before advancement.
55.			Distance to Radiotherapy Centre	Socioeconomic Status						In this study, we discovered that BC patients at one medical facility in Kenya experience, on average, more than a 3-month delay between symptom onset and presentation, more than a 4-month delay between presentation and diagnosis, and a 1.5-month delay between diagnosis and initial treatment. Primarily socioeconomic status, health history factors, and geographic barriers contribute to these delays.Misdiagnoses, misinformation, and inadequate health system resources also contributed to BC treatment delays. This emphasizes the need for not only patient education, but also increased awareness among healthcare providers, which could contribute to a BC diagnosis at an earlier stage when the disease is more amenable to treatment and has better long-term outcomes.
56.	Treatment Centre Characteristics	Waiting Times	Distance to Radiotherapy Centre	Socioeconomic Status	Cultural Beliefs	Patient preferences		Lack of Understanding/Awareness		In Rwanda, longer travel distance to tertiary facilities, but not HCs, is associated with late-stage presentation. Optimizing travel distance through decentralized diagnostic testing or transport subsidies should be prioritized in early detection interventions.
57.	Treatment Centre Characteristics			Socioeconomic Status						There is currently a substantial burden of CRC in Tanzania that appears to be increasing over the past 10 years. While there is capacity in the country to provide

										all aspects of the multidisciplinary care required to treat CRC patients, late presentation, limited access to diagnostic and treatment services and poor coordination continue to be significant barriers to providing optimal treatment to these patients. Recently, the Tanzanian Surgical Association hosted a CRC symposium aimed to update clinicians on the practice guidelines for the treatment of CRC and discuss opportunities to implement system-wide interventions to improve the care of patients with colon cancer in Tanzania. Through this symposium, a multidisciplinary working group convened focused on improving the care of CRC patients within the country.
58.	Treatment Centre Characteristics			Socioeconomic Status	Cultural Beliefs	Patient preferences		Lack of Understanding/Awareness		
59.			Distance to Radiotherapy Centre		Cultural Beliefs					Geospatial access to a tertiary care facility is independently associated with stage at presentation and overall survival among patients with breast cancer in Nigeria. Addressing disparities in access will be essential to ensure the development of an equitable health policy.
60.			Distance to Radiotherapy Centre	Socioeconomic Status						This study identified two potential factors, travel time and HIV status, that influence access to comprehensive cervical cancer care in Botswana.
61.			Distance to Radiotherapy Centre	Socioeconomic Status	Cultural Beliefs	Patient preferences				In conclusion, increasing education and awareness among women, both women living with and without HIV, and among providers, including traditional healers, about the benefits of cervical cancer screening and about the importance of seeking prompt medical care for abnormal vaginal bleeding, while also developing support systems for unmarried women,

										may help reduce cervical cancer morbidity and mortality in Botswana. Moreover, focusing future interventions on education, screening, and referral infrastructure for cervical cancer in areas of Botswana where fewer women are presenting for cervical cancer care, may increase the impact of programs and decrease the cervical cancer burden. Lastly, identifying the need to investigate barriers to accessing advanced cancer care including the travel burden, cervical cancer awareness, knowledge, and resources to ensure equitable access to appropriate treatment for all women in Botswana, both WLWH and without, is needed to improve cervical cancer outcomes.
62.				Socioeconomic Status						This positive stage distance association held to 40 km, and plateaued or slightly reversed in patients (9%) living beyond this distance. Studies of woman and the societal and healthcare-level influences on these delays and on the late stage at diagnosis distribution are needed to inform interventions to improve diagnostic stage and breast cancer survival in this and similar settings.
63.	Treatment Centre Characteristics		Distance to Radiotherapy Centre		Cultural Beliefs			Lack of Understanding/Awareness		In conclusion, the present results provide up-to-date empirical data on woman's access to breast cancer treatment in SSA, and on the key role played by major financial and some sociocultural barriers. The marked between-country divide in treatment access, coupled with the marked and consistent within-population socio-economic differentials in the proportion of untreated patients, supports the call for universal free access to cancer diagnosis and treatment in SSA to prevent

							growing social inequities in breast cancer care and survival in the region.
64. Treatment Centre Characteristics	Distance to Radiotherapy Centre		Patient preferences		Lack of Understanding/Awareness		The present study provides evidence that geographical distance to cancer care services is a barrier to early diagnosis of breast cancer in SSA, indicating potential avenues for downstaging this disease. Potential approaches to reducing this barrier include providing transport or travel allowance to women, particularly those living far away from the hospitals, or considering decentralizing diagnostic services in conjunction with accelerated referral and follow-up. Population-specific interventions will be needed to develop targeted approaches to tackle these barriers to early diagnosis of breast cancer.
65.	Distance to Radiotherapy Centre						In summary, our findings show that in European countries, travel distance from home to retinoblastoma centre is not a barrier to early disease diagnosis. European patients travel on average more than 400 km and >60% present at stage cT2 or earlier. In Africa, the picture is more complex patients travel on average less than 200 km, yet >80% present at stage cT3 or worse, suggesting that factors other than geographic distance to retinoblastoma centre play a role in late disease diagnosis. Poor awareness and education by both caregivers and health workers, other barriers to access, and possibly, number and distribution of specialist retinoblastoma treatment centres in those African countries in which the population is underserved, are key factors that warrant intervention on national and international levels. Familial retinoblastoma is more common in Europe

										than in Africa, most probably due to death related to late disease presentation, and screening of patients at risk of developing retinoblastoma is more common in Europe. Comprehensive counselling of families and patients with germline disease (ie, bilateral retinoblastoma and/or positive family history) may be found useful in order to detect the disease at early stage to increase survival rates in this highly curable malignancy.
66.	Treatment Centre Characteristics	Waiting Times	Distance to Radiotherapy Centre	Socioeconomic Status						In summary, we find that differences in health system delay and advanced stage among male compared to female cancer patients in Botswana appear to be mainly driven by differences in the types of cancers that arise within each sex. Interventions that raise awareness of cancer symptoms among men and sensitization of health providers to cancers common in men could help reduce the length of time between first clinic visit with oncology related system and treatment initiation for all cancer patients, and hopefully improve prognosis for male patients in Botswana. Future studies should explore the role of patient beliefs and health system factors on delays and stage at presentation.
67.	Treatment Centre Characteristics			Socioeconomic Status						In this cross-sectional study of geographic accessibility and availability of radiotherapy, Ghana had major national deficits of radiotherapy capacity, with significant geographic disparities among regions. Well-planned infrastructure scale-up that accounts for the population distribution could improve radiotherapy accessibility.
68.	Treatment Centre			Socioeconomic Status						This study provided an in-depth comparative description by oncology site

	Characteristics									of urban and rural patient sociodemographic and clinical characteristics, pathways to breast cancer diagnosis and treatment approaches. Findings may inform policy interventions to redress patient access barriers and inequities in service resources and infrastructure.
69.	Treatment Centre Characteristics									The results indicate a continuous annual increase in cancer patients treated by radiotherapy in Kenya radiotherapy centers. The increase may be attributed to the rising population, limited access to cancer awareness, and the growing adoption of unhealthy lifestyles, among other factors. Female cervical and breast cancer patients contracted the disease at younger ages (46 - 50 years) compared to the male prostate cancer patients with a mean age of 61 - 65 years. Socio-economic factors, the organization of healthcare systems, and a limited workforce have been identified as some of the barriers to the provision of proper radiotherapy services in the country.
70.	Treatment Centre Characteristics		Distance to Radiotherapy Centre							The results of this study show that health system and its organization present barriers to access of cervical cancer treatment and care among women. Strong political will, mobilization of resources both domestically and from partners in addition to sound policies are imperative to address key health system challenges.
71.			Distance to Radiotherapy Centre	Socioeconomic Status				Lack of Understanding/Awareness		There are numerous prevailing multi-dimensional barriers to accessing cervical cancer treatment and palliative care in a low-income setting. The findings of this study revealed that health system and societal factors were more important than individual level factors. Multi-sectoral

										approaches are recommended to address all the multifaceted barriers in order to improve cervical cancer treatment and palliative care access for better outcomes in resource-limited contexts.
72.			Socioeconomic Status	Age						In summary, the ultimate aim of this study is to target radiation therapy technology developments to produce a robust LINAC that is capable of performing well in challenging environments, such as those encountered in many LMICs, and that will require fewer qualified experts for routine operation and maintenance, especially those personnel who are currently lacking in LMICs. The detailed analysis of the information from this study that will be reported later will complement general LINAC design considerations that include well-recognised factors such as ease of operation, reliability, robustness, easy repairability, self-diagnosis of subsystem faults, insensitivity to power interruptions, lower power requirement, reduced heat production and easy upgradability.
73.		Distance to Radiotherapy Centre								There have been significant advancements being made in screening, workup, and management of patients with cervical cancer in sub-Saharan Africa; yet, improvement is still needed. Enrollment in clinical trials remains a struggle. Participants would like to enroll patients on clinical trials with Cervical Cancer Research Network's continuous support.
74.	Treatment Centre Characteristics									Prior measures of radiation therapy access provide an incomplete picture. Geographic location of radiation therapy centers is a crucial component of access that should be considered for future planning in SSA.
75.	Treatment	Distance	Socioecono				Lack	Lack of		The findings of the survey have enabled

	Centre Characteristics		to Radiotherapy Centre	mic Status			of Referral	Understanding/Awareness		the identification of gaps in cancer control. The above findings suggest that to sustainably improve access to cancer care and treatment, a wide range of interventions targeting the major arms of cancer care and treatment will be required. The Government of Cameroon has demonstrated good political will in this line and has translated the recommendations into actionable interventions for implementation. This report complements the 5-year national strategic plan for prevention and cancer response and enhances the endeavour of the Ministry of Health.
76.				Socioeconomic Status						In Africa, despite the growing burden of CNS tumors, access to neuro-oncology care remains suboptimal due to various factors, including a shortage of skilled professionals, inadequate infrastructure and resources, and a lack of public awareness regarding brain tumors. To address these challenges, there is a need to implement initiatives that raise public awareness and provide education, as well as establish specialized mobile clinics equipped with essential medical equipment."
77.	Treatment Centre Characteristics	Waiting Times	Distance to Radiotherapy Centre							In summary, there are considerable challenges to meeting the demands of the impending cancer crisis in Africa. These include costs for equipment and maintenance as well as deficits in human resources and training. Addressing these issues will be crucial to tackling the increasing burden of cancer on the continent. Innovations in technology and collaborative efforts within the global oncology community can identify sustainable paradigms for establishing

										quality cancer care in Africa and eradicating the massive disparities that currently exist.
78.	Treatment Centre Characteristics	Waiting Times	Distance to Radiotherapy Centre	Socioeconomic Status	Cultural Beliefs	Patient preferences				The resurrection of Gabonâ€™s radiation therapy services is a relative success story considering the current situation whereby multiple African nations are struggling to provide essential radiation therapy services to their populace. The revival of Gabon's radiation therapy service involved tripartite efforts between the Gabonese government, IAEA, and the NGOs spearheaded by Lalla Salma and Sylvia Ondimba Bongo, the first ladies of Morocco and Gabon. This collaboration is a model of cooperation worth emulating to rapidly develop the radiation therapy sector on the African continent. However, it is important to immediately address current needs such as provision of a brachytherapy machine and regular machine maintenance to prevent regression and loss of the current gains.
79.			Distance to Radiotherapy Centre							We identify common threads across regions, including sparse distribution of radiation equipment, geographic access, and specialized training. We also highlight examples of success in the use of telemedicine and cross-cultural partnerships to help bolster access to training to ensure increased access to adequate and appropriate treatment of gynecologic malignancies.
80.			Distance to Radiotherapy Centre	Socioeconomic Status						Promoting access to high-level treatment requires that national health researchers and policymakers have access to information about the global investment needed to ensure adequate cervix cancer therapeutics . The development of health data systems linking treatment

						information to cancer registries and death registries may be an important step to better recognize the impact of quality of care on patient outcome. Large database generated from such registries are particularly relevant to monitor the impact of health care policies, the benefit of therapeutic innovation, and the beneficial consequences of increasing compliance to modern standards. Medico-economic evaluations are also required to sensitize decision makers to the real benefit of increasing quality of cervical cancer care (e.g. implementation of image-guided brachytherapy), for patients but also for society. In addition to vaccination and early screening, increasing accessibility to high-quality treatments should be a complementary priority. This implies strengthening investments into imaging modalities, surgery, radiotherapy, brachytherapy and best supportive care to achieve this objective.
81. Treatment Centre Characteristics			Lack of Referral			Dissemination and implementation of resource-stratified guidelines, addressing social and cultural barriers, increasing awareness and early detection, encouraging advocacy, optimizing medical and surgical oncology training and reducing brain drain phenomenon, improving international exchanges of knowledge and multidisciplinary management, facilitating access to modern drugs and therapy, in addition to increasing physical and human resources are all needed to improve access to care and to reduce disparities in cancer patients outcome worldwide. Partnering of international organizations with LMICs may prove a promising way to improve

											quality cancer care in LMICs.
82.				Socioeconomic Status							If Nigeria is to arrest this decline in the provision of quality radiation therapy, then those in charge of health policy must direct their efforts toward achieving universal health coverage, plan realistically for national needs based on national data, create an enabling environment for quality radiation oncology training and practice, curb corruption, and guarantee the national electricity grid and power supply. There must be continual efforts to acquire and add new machines and maintain existing machines until the national demand is met, lest the biblical saying be fulfilled: â€œfor whosoever hath, to him shall be given; and whosoever hath not, from him shall be taken even that which he seemeth to have.
83.	Treatment Centre Characteristics		Distance to Radiotherapy Centre	Socioeconomic Status					Lack of Understanding/Awareness		Developing quality diagnostic services in the proper health context is crucial for early diagnosis and successful therapy of CRC patients, and applying a resource-sensitive approach to prioritize essential treatments based on effectiveness and cost-effectiveness is key to overcoming barriers in LMICs, with clinical research collaborations between high-income countries (HICs) and LMICs being a helpful strategy to improve health indicators and prevent the burnout of health workers.
84.		Waiting Times	Distance to Radiotherapy Centre								In conclusion, radiotherapy for gynecological cancers in sub- Saharan Africa is a significant example of the need for a holistic development approach across different sectors of the economy and different disciplines of medicine. The complexity of its management continues to expose the underdevelopment of health and financial systems as well the lack of

										universal health coverage and social systems as we continue to see unnecessary morbidity and mortality due to the lack of organization. More systematic and scientifically robust investigations tailored to the various sub-Saharan African countries need to be conducted to elicit disruptive local solutions to the status quo.
85.		Waiting Times	Distance to Radiotherapy Centre							PCa in Africa is not just a medical problem Integrative research on elucidating how western PCa healthcare practices can be integrated into African cultural and traditional practices towards PCa management is warranted. In Africa, factors leading to poor PCA management are interconnected. This calls for collaborative efforts to effectively manage this disease.
86.		Waiting Times								Senegal doesn't have good statistics on cancer numbers or deaths. Currently, Diop says, prostate cancer is thought to be the country's number one cancer. But he is convinced that breast cancer is a bigger killer in Senegal.
87.	Treatment Centre Characteristics	Waiting Times		Socioeconomic Status						Lung cancer is of public health importance with a great degree of socioeconomic burden. Case fatality rate is higher than any other commonly reported cancer. Late presentation is a major factor to the high fatality rate. Well-coordinated and regularly supervised awareness and education campaign championed by the Nigerian Thoracic Society and similar national associations across the West African subregion could help. Campaigns should target the first-line health-care practitioners on the need for early recognition and referral to the appropriate specialist centers.

88.	Treatment Centre Characteristics	Waiting Times	Distance to Radiotherapy Centre	Socioeconomic Status	Cultural Beliefs			Lack of Understanding/Awareness		In 2016, the means to prevent and treat cervical cancer are well known and widely available; a death from cervical cancer should be understood as a preventable and unnecessary death. The benefit from vaccination programs will not be realized for decades, leaving millions of women at risk. In areas with limited medical resources, programs of primary and secondary prevention can significantly decrease the burden of cervical cancer. Complete and comprehensive cervical cancer control, however, requires a broadly coordinated effort from multiple specialists and facilities. These specialists can only be trained, and such care can only be safely given, in the setting of a strong overall health system. We propose that outreach efforts in cervical cancer control should broaden their targets beyond process-based and disease-based metrics and work to more broadly strengthen the overall health system.
89.	Treatment Centre Characteristics	Waiting Times		Socioeconomic Status	Cultural Beliefs			Lack of Understanding/Awareness		In conclusion, education awareness campaigns, uplifting of socioeconomic conditions, access to diagnostic resources, and availability of higher standards of health care, and sensitivity with regard to some patients beliefs are all necessary, should with regard to some patients beliefs are all necessary, should be implemented, and must be considered in an attempt to increase early detection of breast cancer and, therefore,improve long-term prognosis and survival. This is a challenge to be fulfilled mainly in the rural and disadvantaged populations, but campaigns should be extended challenge to be fulfilled mainly in the rural and disadvantaged populations, but

											campaigns should be extended to all women, irrespective of their race, socioeconomic and cultural status, and place of residence.
90.	Treatment Centre Characteristics		Distance to Radiotherapy Centre	Socioeconomic Status	Age						In conclusion, there are substantial variations in geographic access to cancer care in Rwanda. GIS modeling methods can be a valuable tool for describing the magnitude of this variation and for highlighting cancer care access inequity in vulnerable populations. These methods also have potential for influencing planning of national care expansion and CCCs to Rwanda referral hospitals would meaningfully improve access to cancer treatment for all Rwandans and decrease care inequities especially faced by vulnerable populations in rural and poor areas.
91.	Treatment Centre Characteristics		Distance to Radiotherapy Centre								Developing countries continue to bear a disproportionate percentage of the global cervical cancer burden. Investigations into the growing gap in incidence and mortality between developed nations and LMIC have cited persistent financial, infrastructural and educational limitations as key drivers. Pervasive lack of access to both preventative and definitive care has left a substantial portion of cervical cancer patients with minimal options for disease management. For countries with little to no radiotherapy capacity, few evidence based recommendations exist to guide therapeutic decision making. Settings with brachytherapy but not EBRT are currently considered equivalent to those without any radiotherapy units, according to the most recent ASCO guidelines. Here, we argue that historical data supports the use of brachytherapy alone in cervical cancer.
